# Investigation of the effect of virtual reality glasses used during extracorporeal shock wave lithotripsy on patients’ pain, anxiety, and comfort levels: a randomized controlled trial

**DOI:** 10.1007/s00240-026-02018-w

**Published:** 2026-06-15

**Authors:** Seda Cansu Yeni̇ğün Akbulut, Seval Ulubay

**Affiliations:** 1 Department of Surgical Disease Nursing, Akdeniz University Kumluca Faculty of Health Sciences, Antalya, 07350 Turkey; 2 Department of Quality Management Unit, Samsun Gazi State Hospital, Samsun, Turkey

**Keywords:** Anxiety, Comfort, Pain, Surgical nursing, Virtual reality

## Abstract

Pain and anxiety are frequent issues that can significantly reduce patient comfort during extracorporeal shock wave lithotripsy. Virtual reality has recently gained attention as a non-pharmacological approach to improve the patient experience in various clinical procedures. This study was conducted to examine how the use of virtual reality during extracorporeal shock wave lithotripsy influences patients’ perceived pain, anxiety, and comfort levels. This single-blind randomized controlled trial was conducted between February 1, 2025, and September 1, 2025. Patients in the virtual reality group received the standard protocol supplemented with virtual reality glasses throughout the procedure, whereas those in the control group received only standard care during the procedure. Study data were obtained through the “Descriptive Information Form,” the “Visual Analog Scale,” the “Visual Analog Scale-Anxiety,” and the “State-Trait Anxiety Inventory.” (NCT06804928). At baseline, the VR and control groups were homogeneous with respect to descriptive variables (*p* > 0.05). Following ESWL, pain scores increased in the control group, whereas a significant reduction was observed in the VR group, while comfort scores improved significantly only in the VR group (*p* < 0.05). Anxiety levels showed no significant change in the control group but were significantly reduced in the VR group (*p* < 0.05). Between-group comparisons confirmed lower post-procedure pain and anxiety and higher comfort in the VR group (*p* < 0.05). This study revealed that the use of virtual reality glasses during ESWL reduces pain and anxiety levels and enhances patient comfort, suggesting that VR may serve as a promising and feasible non-pharmacological method in clinical nursing practice.

## Introduction

The prevalence of urinary stones has shown a significant increase over the years. While the prevalence was 3.8% in 1970, it rose to 8.8% in 2010 and reached 11% in the United States alone by 2022 [1]. Each year, it is estimated that more than one million individuals visit emergency departments as a result of acute renal colic and nephrolithiasis, with nearly one-fifth of the affected individuals necessitating inpatient treatment [2, 3]. As a minimally invasive procedure, ESWL reduces the need for invasive surgery by breaking kidney and ureteral stones into smaller, passable fragments using high-energy pressure waves. Consequently, it reduces hospitalization rates, improves patient outcomes, and alleviates the economic burden associated with urolithiasis [1]. However, pain and anxiety are among the most frequently reported issues experienced by patients following ESWL [5]. Evidence from earlier studies suggest that patients presenting with elevated anxiety levels experience more pain during the procedure [6, 7]. Alleviating pain and anxiety during ESWL is crucial not only for maintaining patient comfort and satisfaction but also for minimizing patient movement during the procedure, which facilitates the visualization and targeting of the stone [8]. Therefore, pharmacological approaches (including general and spinal anesthesia, sedative agents, opioids, or analgesics) are frequently employed [9]. However, despite their effectiveness, these treatments are not recommended due to their high cost and potential side effects such as respiratory depression, hypotension, and allergic reactions [5]. Non-pharmacological methods implemented by nurses offer advantages such as reducing patients’ need for analgesics and anxiety levels, enhancing activity and adaptability, having fewer side effects, and being applicable independently in many cases [10, 11]. In recent years, various non-pharmacological approaches such as massage, hot/cold application, relaxation exercises, acupuncture, acupressure, transcutaneous electrical nerve stimulation, hydrotherapy, and distraction techniques (listening to music, watching films, guided imagery, humor, laughter, stress ball use) have been reported to be effective in reducing pain and anxiety [7, 12]. One of these distraction-based non-pharmacological methods is virtual reality (VR) applications. VR is an immersive technology that creates three-dimensional artificial environments using computer technology, allowing individuals to feel as if they are part of that environment. The literature emphasizes VR as an effective, cost-efficient, and practical tool in physical rehabilitation, pain and anxiety management, and surgical training [13]. While existing studies have examined the impact of non-pharmacological methods on pain and anxiety during ESWL, no study has been found that holistically addresses the use of virtual glasses with patient comfort. Therefore, to fill this gap in the current literature, this study investigates the effects of virtual glasses use during ESWL on pain, anxiety, and comfort.

## Aim

This study was conducted to evaluate the impact of using virtual reality glasses on pain, anxiety, and comfort levels during the ESWL procedure.

## Materials and methods

### Design

**Fig. 1:**
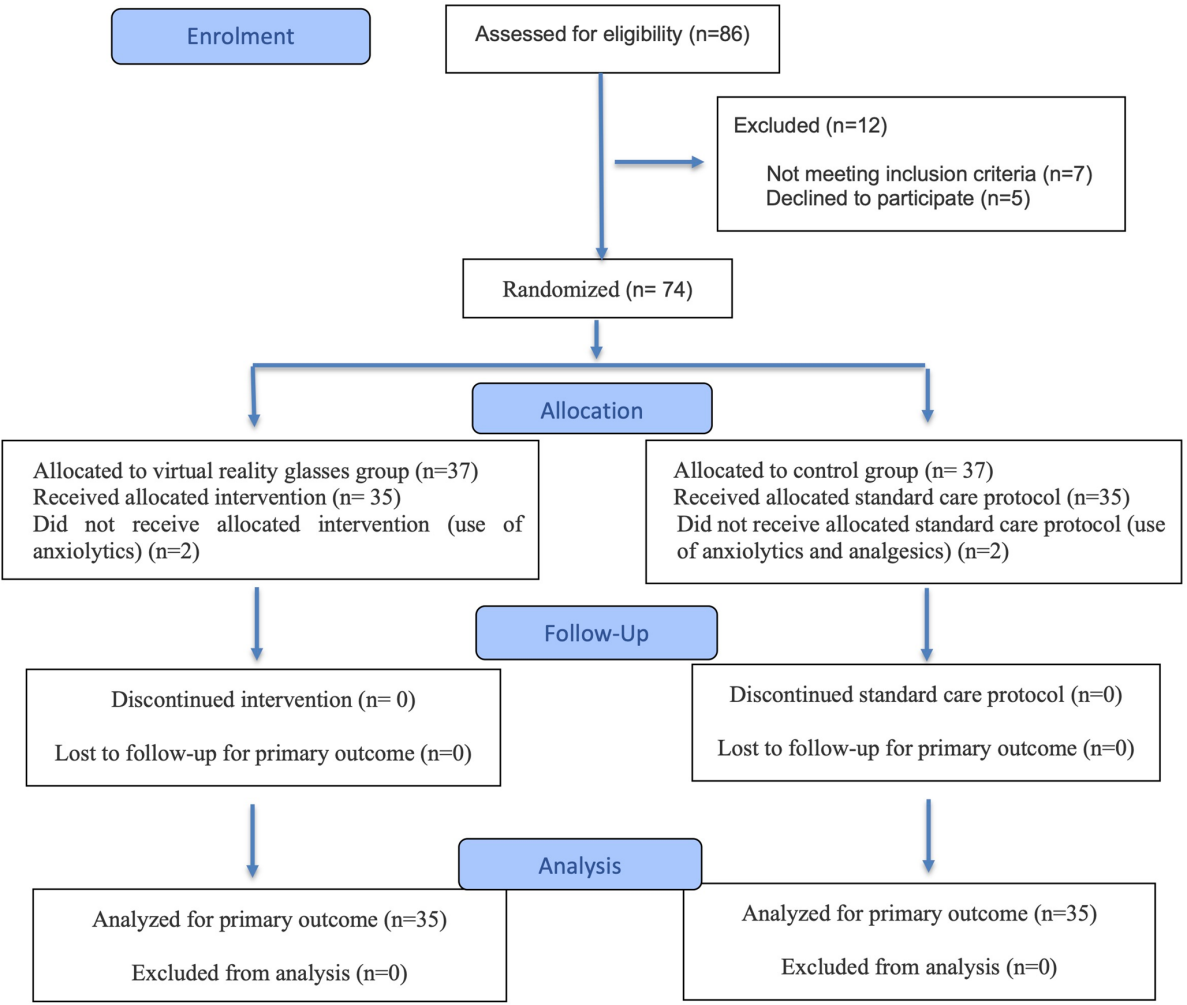
CONSORT 2025 flow diagram

This was a single-blind, two-arm, parallel-group randomized controlled trial conducted in patients undergoing ESWL. Eligible participants who consented were randomly assigned to the virtual-reality glasses group (intervention group) or control group. The trial was conducted and reported in accordance with the Consolidated Standards of Reporting Trials (CONSORT) guidelines [14]. The protocol was registered with the ClinicalTrials.gov Protocol Registration system (NCT06804928). The flow of participants throughout the study is presented in the CONSORT diagram (Fig. 1).

## Study hypotheses

### H_0_

The application of virtual reality glasses during the ESWL does not produce a statistically significant change in the levels of pain, anxiety, and comfort experienced by patients.

### H_1_

The use of virtual reality glasses during the ESWL results in a statistically significant change in patients’ levels of pain, anxiety, and comfort.

## Participants and setting

The study population comprised patients who were admitted for ESWL between February 1, and September 1, 2025, at the day surgery unit of a state hospital located in the Black Sea Region. The sample size was determined using G*Power software (version 3.1.9.7, Heinrich Heine University, Düsseldorf, Germany). Based on a large effect size, based on a large effect size (d = 0.80), a significance level (α) of 0.05, and a statistical power (1–β) of 0.90, the minimum required number of participants was calculated as 34 participants per group with an equal allocation ratio (N2/N1 = 1). Considering a possible 10% attrition rate, 37 patients were recruited for each group, yielding a total of 74 participants. Four patients were excluded after randomization due to the use of anxiolytics and/or additional analgesics, and the final analysis was performed on 70 participants.

Inclusion criteria were as follows: (i) being 18 years of age or older, (ii) scheduled for elective ESWL due to renal or ureteral calculi, (iii) being conscious and able to communicate, (iv) voluntarily participated in the study after providing written informed consent, (v) had negative urine culture results prior to the procedure, (vi) absence of coagulopathy or bleeding disorders, and (vii) having a single radiopaque renal or ureteral calculus measuring ≤ 10 mm in diameter. Exclusion criteria included: (i) visual or auditory impairments preventing the use of virtual reality glasses, (ii) psychiatric or neurological disorders that could interfere with perception of the intervention, (iii) a diagnosis of epilepsy, (iv) the presence of multiple renal or ureteral calculi, (v) abdominal arterial aneurysm, urinary diversion or urinary obstruction, and (vi) the use of any sedatives or anxiolytics before or during the procedure.

## Standard care protocol

Prior to the ESWL procedure, the patient first presents to the outpatient clinic by appointment and undergoes a detailed examination by a physician. The physician evaluates clinical factors such as the patient’s general health status, the size of the stone, and its location. Additionally, a KUB (kidney ureter bladder) X-ray is performed prior to the procedure to confirm that stones are radio-opaque and to verify their anatomical location. Following the examination, necessary laboratory tests are ordered, including blood tests and urine culture samples. The urine culture is particularly important for detecting urinary tract infections, as the presence of infection before the procedure significantly increases the risk of serious complications. After providing the urine sample, the patient is referred to the ESWL unit. On the day of the procedure, the first point of admission is the day surgery unit, where the patient is admitted and given a wristband for identification to ensure accurate patient tracking throughout the procedure. This step is critical for patient safety and identity verification. An intravenous (IV) line is then established, and 500 ml of 0.9% sodium chloride solution containing one ampoule of ketoprofen is administered to all patients prior to the procedure for analgesic purposes. No sedation was administered to the patients before or during the procedure.

All patients receive outpatient care, and lithotripsy procedures are performed using a lithotripter system . For the localization of both kidney and ureteral stones, patients are positioned in the supine position, and the stone location is determined using ultrasound and fluoroscopy. All sessions are carried out under the supervision of a urologist by a nurse with 10 years of experience in the field. Patients with renal pelvis or ureter stones receive a total of 2,500 shock waves delivered at a rate of 60 shocks per minute, with energy levels ranging from 12 to 14 kV. If sufficient stone fragmentation is observed before the maximum number of shocks defined in the protocol is reached, the ESWL treatment is terminated. In this study, none of the patients required the full 2,500 shocks, as adequate fragmentation was achieved before reaching the maximum limit.

After the ESWL procedure, the patient is monitored in the day surgery unit for approximately 1–2 h. During this time, vital signs (pulse, blood pressure, respiration) are monitored every 15 min. Additionally, the patient is closely observed for any potential complications, such as pain, bleeding, nausea, vomiting, or other adverse effects that may occur during or after the procedure. A follow-up outpatient clinic visit is scheduled one week after the procedure to assess renal function and evaluate the fragmentation of the stone. At this follow-up, the patient’s recovery status, procedural success, and any complications are thoroughly reviewed. Upon discharge, patients were routinely prescribed paracetamol or ibuprofen for pain management. In cases of ureteral calculi, an alpha-blocker (tamsulosin 0.4 mg/day) was also prescribed to facilitate stone passage. All patients were advised to maintain adequate fluid intake and monitor their urine output during the post-procedural period.

### Randomization and blinding

The study was conducted using a 1:1 individual-level random assignment into two arms: the intervention group (virtual reality headset + standard care protocol) and the control group (standard care protocol only). The randomization list was generated by an independent statistician, who was not part of the research team, using a computer-based random number generator. Participants could not be blinded due to the nature of the intervention. However, the clinician administering the intervention and the individual collecting the data were independent researchers and were blinded to group assignments. Furthermore, the assessors responsible for gathering all primary and secondary outcomes (pain, anxiety, and comfort), as well as the biostatistician conducting the statistical analyses, were also blinded to group allocation. Group information in the data collection forms was coded, without revealing actual group names. To evaluate the integrity of the blinding, assessors were asked to guess the group assignment after the analysis was completed.

## Data collection

### Descriptive information form

This form was developed by the researchers based on the relevant literature [15–17]. The Patient Information Form consists of 14 questions addressing patients’ socio-demographic characteristics (age, gender, level of education, marital status, number of children, and employment status, alcohol and tobacco use, presence of chronic disease) and clinical information related to the illness (regular medication use, previous hospitalization history, history of surgery, information about ESWL, and duration of lithotripsy).

### State-Trait Anxiety Inventory (STAI)

The scale, developed by Spielberger et al. (1970) [18] and adapted into Turkish with validity and reliability analyses conducted by Öner and Le Compte (1985) [19], consists of 40 items. It is composed of two subscales: the first part, the State Anxiety Inventory, includes 20 items measuring the individual’s emotional state at a specific moment; while the second part, the State-Trait Anxiety Inventory, also includes 20 items and assesses the individual’s overall tendency to experience anxiety. Each subscale yields scores ranging from 20 to 80, with higher scores reflecting greater levels of anxiety. In the present study, the Trait Anxiety Inventory, which evaluates persistent anxiety, was employed. Within the scope of the study, the similarity of patients’ pre-procedural trait anxiety levels was considered an important variable.

### Visual Analog Scale (VAS)

The scale was originally introduced by Price et al. in 1983 [20]. In recent years, it has been commonly employed to evaluate patients’ subjective perceptions of pain and comfort. It consists of a 10-cm line placed either horizontally or vertically, with the two endpoints indicating the extremes of the construct being measured: “0” corresponds to “no pain/no discomfort (not at all comfortable)” and “10” corresponds to “severe pain/very uncomfortable (very comfortable) [21]. Patients described comfort as both freedom from physical unease and the presence of pleasant sensations. To obtain the scores, researchers asked participants to indicate their pain and comfort level by placing a handwritten mark along the 10-cm line. In the literature, it has been reported that the Visual Analog Scale (VAS) has been used not only for pain assessment but also for evaluating patient comfort during surgical interventions [22, 23].

### Visual Analog Scale for Anxiety (VAS-A)

The validity of this scale for anxiety assessment was confirmed by Davey et al. (2007) [24]. On the scale, the left endpoint represents “0 = no anxiety”, while the right endpoint indicates “10 = extremely high anxiety.” Participants were instructed to mark the point on the line that best reflected their perceived level of anxiety. Evidence from the literature has demonstrated that the VAS-A is a valid and reliable instrument for evaluating anxiety levels during surgical procedures [12, 15].

### Study procedure

This study was conducted in two phases. The first assessment was conducted before the patient underwent the ESWL procedure, and the second assessment was carried out 30 min after the procedure. As the study followed a single-blind randomized controlled design, the patient information form used during the first assessment was completed by a specialized research nurse. Pain, anxiety, and comfort levels, measured using the VAS scale before and after ESWL, were assessed by a nurse with six years of experience working in the unit. The State-Trait Anxiety Inventory-Trait (STAI-T) was administered only once, prior to the ESWL procedure.

### First stage

After obtaining ethical approval and institutional permissions for the study, the expert research nurse visited the clinic, introduced herself to the patients admitted for ESWL, and informed about the purpose of the study. Written informed consent was obtained from patients both in written and verbal form. Socio-demographic data were gathered through face-to-face interviews utilizing the patient information form, while clinical data were retrieved from the patients’ medical records. Then, the unit nurse assessed patients’ state anxiety, pain, anxiety, and comfort levels using the VAS scale. This process took approximately 10–15 min. The first phase of data collection was conducted preoperatively in the waiting area before the ESWL procedure.

### Second stage

#### Virtual reality group

After the first assessment, the expert research nurse visited the patient’s room and informed them that they would be watching a video through virtual reality goggles during the procedure. The patients were instructed to wear the goggles throughout the procedure and were told that the nurse would be present beside them throughout, and that they could remove the goggles at any time if needed. To prevent any adverse effects during the intervention Universal Headrest Pad was placed under the patient’s head while lying in a supine position, allowing them to rest with their head turned to the side.

In the intervention group, patients used the Meta Quest 2 VR-G (Meta Platforms Technologies, Inc., Menlo Park, CA, USA) virtual reality headset. The Meta Quest 2 VR headset can be paired with compatible smartphones via the mobile application and Bluetooth connection. Its design resembles that of the original Quest but features a white plastic exterior instead of the black fabric covering. At 503 g (17.7 oz), it is lighter than the first-generation Quest (571 g, 20.1 oz). Additionally, the elastic strap of the first model was replaced with an adjustable Velcro fabric strap. After each use, the headset was disinfected to ensure hygienic conditions.

**Fig. 2:**
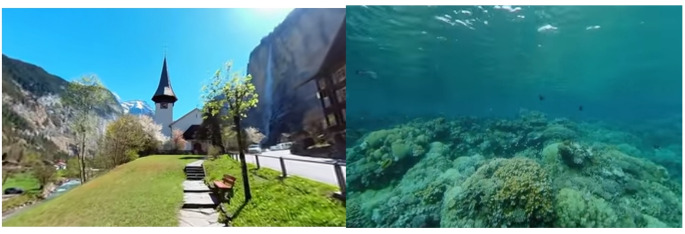
Comparison of pain levels before and afterthe *ESWL *procedure between the control group and the virtual reality glasses group

The videos used for individuals in this group consisted of two titles: “Lauterbrunnen: Close to Heaven / Switzerland 4K / 360 / VR VIDEO” (13 min and 6 s) [25], and “Underwater Life, Marsa Alam, Egypt. 360 video in 8K” (11 min and 1 s) [26] (Fig. 2).

### Control group

After the first assessment, the expert research nurse returned to the patient and informed them that the procedure would begin. Patients in both groups were taken to a recovery room after leaving the ESWL unit. Thirty minutes after the procedure, their levels of pain, anxiety, and comfort were assessed using the VAS by the unit nurse. This evaluation took approximately 5–10 min.

### Data assessment

The data collected in the study were analyzed using the “Statistical Package for the Social Sciences (SPSS) version 26.0”. Descriptive characteristics of the participants in the virtual reality (VR) group and the control group during the ESWL procedure were presented as frequencies and percentages. Age and measurement-related data were expressed as mean, standard deviation, median, minimum, and maximum values. The normality of data distribution was evaluated through “skewness” and “kurtosis” values, indicating that all numerical variables followed a normal distribution. The acceptable range for normality was set at ± 1.96 [27]. Differences between groups in categorical variables were analyzed using the Chi-square test (χ²). The Paired sample t-test was employed to compare pre- and post-test scores within the Control and VR groups, while the “Independent Samples T-Test” assessed differences between the two groups.

### Ethical considerations

#### Ethical approval

Ethical approval for the study was obtained from the University Non-Interventional Clinical Research Ethics Committee (No: 2025/2/11, date: 24.01.2025). In addition, permission to conduct the research was obtained from the Provincial Directorate of Health. All procedures were conducted in accordance with the principles outlined in the Declaration of Helsinki. Prior to participation, patients were informed about the study and both verbal and written consent were obtained. As the educational video used in the study is publicly available on the YouTube platform, no additional permission was required.

## Results

### Comparison of the descriptive characteristics of patients in the virtual reality group and the control group during the ESWL procedure

A total of 70 patients participated in the study. Of the participants, 35 (50.0%) were assigned to the control group and 35 (50.0%) to the virtual reality group (VRG). The gender distribution was similar between the groups, with 45.7% of the control group and 48.6% of the VR group being female (*p* > 0.05). In terms of education level, the majority of participants in both groups were high school, and no significant differences were found between the groups (*p* > 0.05). Similarly, there were no statistically significant differences between the groups in terms of marital status, employment status, smoking and alcohol use, history of chronic illness, previous hospitalizations, or previous surgeries (*p* > 0.05).

All participants in both groups received information about the ESWL procedure before treatment (100%), and all had social health insurance. Regarding numerical variables, the mean age was 45.6 ± 7.72 years in the control group and 44.6 ± 8.64 years in the VR group. No statistically significant differences were found between the groups in terms of age, smoking duration, lithotripsy duration, or trait anxiety (STAI-T) scores (*p* > 0.05). The results showed that both the virtual reality and control groups were similar in their descriptive characteristics, with no statistically significant differences identified between them (Table 1).

**Table 1:** Comparison of the descriptive characteristics of patients in the group using virtual reality glasses during the eswl procedure and the control group

Variables		Control Group (CG)(*n*:35)		Virtual Reality Glasses Group (VRG) (*n*:35)		
		*n*	%	*n*	%	*p*
Gender	Female	16	45.7	17	48.6	1.000
	Male	19	54.3	18	51.4	
Education	Primary school	8	22.9	10	28.6	0.480
	High school	20	57.1	15	42.9	
	University	7	20.0	10	28.6	
Marital status	Single	8	22.9	11	31.4	0.591
	Married	27	77.1	24	68.6	
Social security	No	0	0.0	0	0.0	-
	Yes	35	100.0	35	100.0	
Employment status	No	12	34.3	9	25.7	0.602
	Yes	23	65.7	26	74.3	
Tobacco use	No	14	40.0	11	31.4	0.618
	Yes	21	60.0	24	68.6	
Alcohol use	No	26	74.3	21	60.0	0.309
	Yes	9	25.7	14	40.0	
Chronic disease	No	18	51.4	22	62.9	0.469
	Yes	17	48.6	13	37.1	
Previous hospitalization	No	19	54.3	16	45.7	0.633
	Yes	16	45.7	19	54.3	
History of previous surgeries	No	28	80.0	24	68.6	0.412
	Yes	7	20.0	11	31.4	
Training on lithotripsy (stone-breaking procedure)	No	0	0.0	0	0.0	-
	Yes	35	100.0	35	100.0	
		*Mean.±S.D.* *Med. (Min.-Max.)*		*Mean.±S.D.* *Med. (Min.-Max.)*		*p*
Age		45.6 ± 7.72 46 (26–61)		44.6 ± 8.64 44 (28–60)		0.611
Duration of smoking (years)		19.38 ± 7.02 19 (8–33)		17.96 ± 7.87 20 (6–35)		0.525
Trait Anxiety (STAI-T)		48.43 ± 6.84 50 (40–60)		50.8 ± 6.82 50 (40–60)		0.151
Trait Anxiety (STAI-T)		51.37 ± 3.68 52 (44–60)		51.17 ± 4.01 51 (44–60)		0.829

Note: . The Chi-square test (χ²) was used for categorical data. and the Independent Samples t-test was used for numerical data.

### Comparison of Pre- and Post-Procedure Pain, Anxiety, and Comfort Levels in the Virtual Reality and Control Groups During the ESWL Procedure

**Fig. 3:**
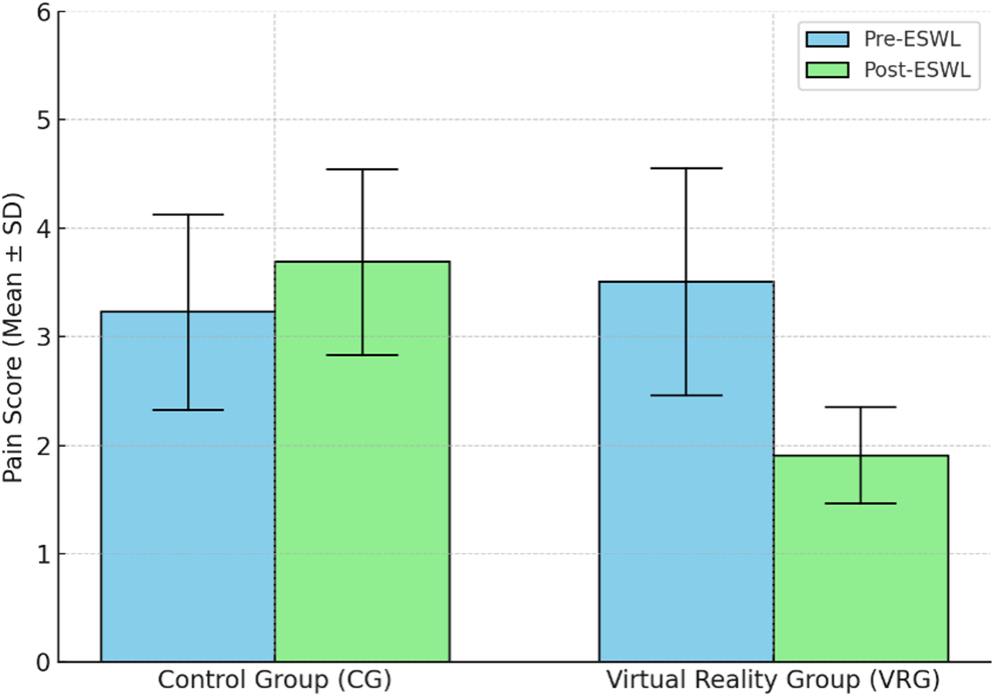
Comparison of comfort levels before and after the ESWL procedure between the control group and the virtual reality glasses group

In the following tables and analyses, p₁ values represent within-group (pre–post) comparisons, whereas p₂ values indicate between-group comparisons. The comparison of pre-and-post-ESWL pain, anxiety, and comfort levels in patients from the virtual reality group and the control group were presented in Table 2. According to the findings, prior to the ESWL procedure, the mean pain score was 3.23 ± 1.50 in the control group and 3.51 ± 1.74 in the virtual reality group, and no statistically significant difference was found between the groups (p₂ > 0.05). However, following the procedure, the pain score increased to 3.69 ± 1.43 in the control group, whereas it significantly decreased to 1.91 ± 0.74 in the virtual reality group. This difference between the groups was statistically significant (p₂ < 0.05). Furthermore, intragroup comparisons demonstrated a significant increase in pain levels in the control group after the procedure (p₁<0.05), while a marked and statistically significant decrease in pain was observed in the virtual reality group post-procedure (p₁<0.05) (Table 2; Fig. 3).

**Table 2:** Comparison of pain anxiety and comfort levels before and after the eswl procedure in patients from the virtual reality glasses group and the control group

Variable	Group	Before ESWL	After ESWL	
		Mean.±S.D.	Mean ± S.D.	*p* ^1^
Pain	Control group* (CG)*	3.23 ± 1.50	3.69 ± 1.43	0.030*
	Virtual Reality Glasses Group* (VRG)*	3.51 ± 1.74	1.91 ± 0.74	0.000**
	*p* ^*2*^	0.464	0.000**	
Comfort	Control Group* (CG)*	5.06 ± 1.64	4.71 ± 1.54	0.258
	Virtual Reality Glasses Group* (VRG)*	4.80 ± 1.66	7.69 ± 0.87	0.000**
	*p* ^*2*^	0.517	0.000**	
Anxiety	Control Group* (CG)*	4.91 ± 2.29	4.66 ± 2.17	0.621
	Virtual Reality Glasses Group* (VRG)*	5.29 ± 2.14	3.09 ± 1.15	0.000**
	*p* ^*2*^	0.486	0.000**	

p < 0.05. *p < 0.01.

*p₁: *Comparison of pre- and post*-ESWL *measurements within each group (Paired Samples* t-*Test).

*p₂: *Comparison of the measurements at each time point between the* CG *and *VRG* groups* (*Independent samples* t-*test).

**Fig. 4:**
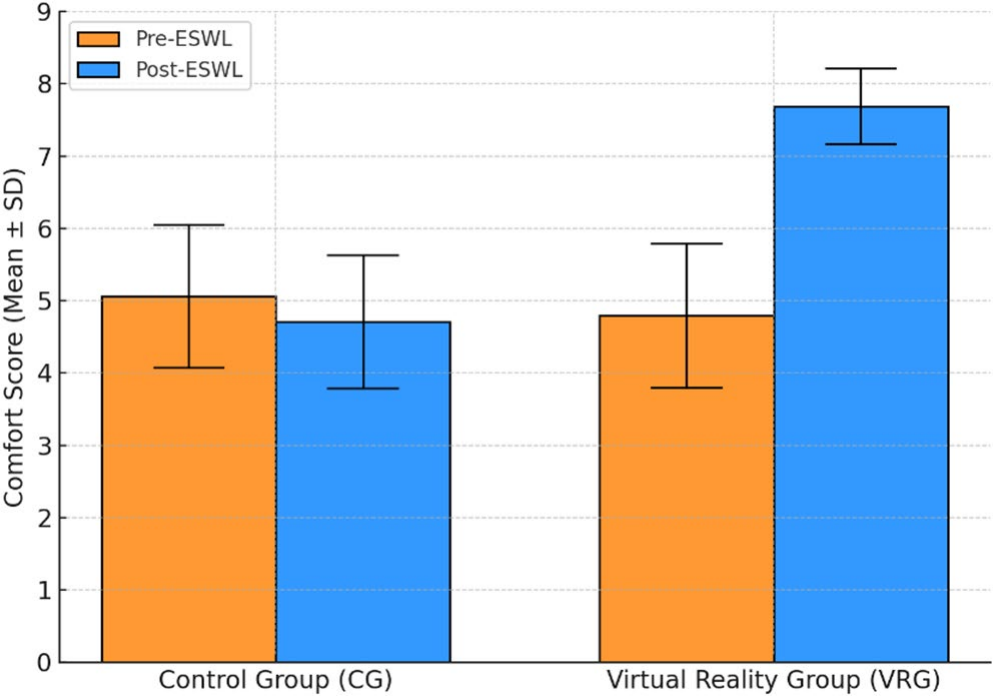
Comparison of anxiety levels before and after the ESWL procedure between the control group and the virtual reality glasses group

Before the ESWL procedure, the mean comfort score was 5.06 ± 1.64 in the control group and 4.80 ± 1.66 in the virtual reality group, with no significant difference observed between the two (p₂ > 0.05). After the procedure, the comfort score in the control group decreased to 4.71 ± 1.54, whereas the virtual reality group showed a statistically significant increase in comfort score to 7.69 ± 0.87 (p₂<0.05). In intra-group comparisons, there was no significant change in comfort levels in the control group before and after the procedure (p₁>0.05), while the increase in comfort level post-procedure in the virtual reality group was found to be highly significant (p₁<0.05) (Table 2; Fig. 4).

**Fig. 5:**
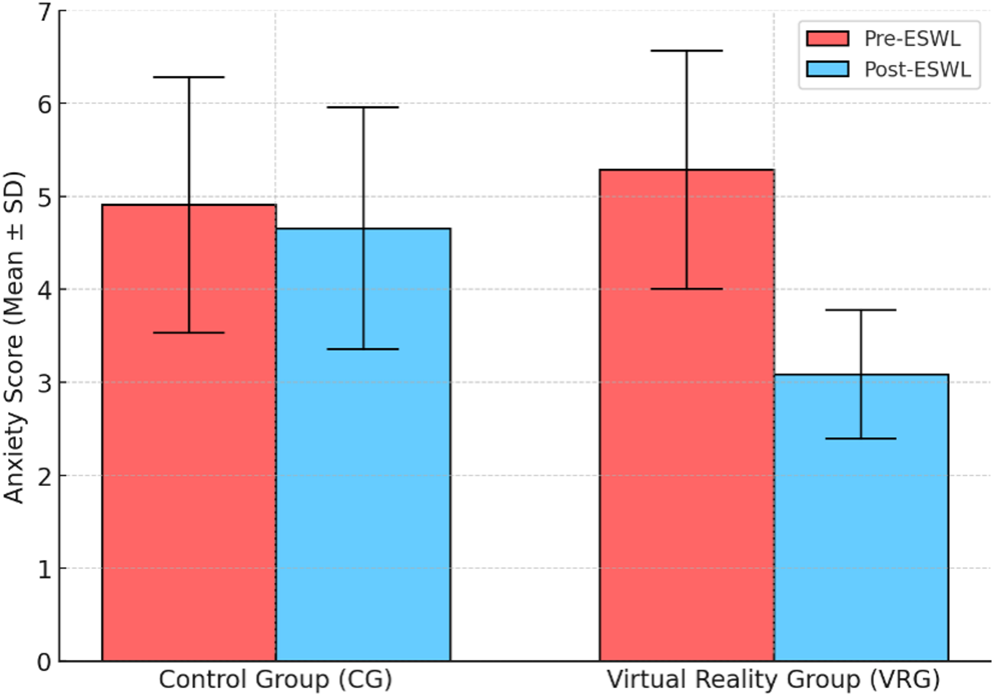
Videos image

Pre- and post-procedure anxiety levels of the control and virtual reality groups were also compared. Before the procedure, the mean anxiety score was 4.91 ± 2.29 in the control group and 5.29 ± 2.14 in the virtual reality group, with no statistically significant difference between the two (p₂ > 0.05). After the procedure, the anxiety score in the control group decreased slightly to 4.66 ± 2.17, but this change was not statistically significant (p₁>0.05). In contrast, the anxiety score in the virtual reality group decreased significantly to 3.09 ± 1.15, and this change was found to be highly statistically significant (p₁<0.05). In the between-group comparison, post-procedure anxiety levels were significantly lower in the virtual reality group than in the control group (p₂ < 0.05) (Table 2; Fig. 5).

## Discussion

Although ESWL is generally an outpatient procedure that does not require anesthesia or sedation, patients may experience both pain and anxiety throughout the procedure [15, 28]. There are multiple factors that may contribute to the occurrence of pain and anxiety. Shock waves can directly affect cutaneous pain receptors, muscle tissue, or skeletal structures such as the ribs, and may also create tension in the renal capsule, leading to significant pain in the patient. In addition, the continuous operation of the ESWL device and prolonged procedure time can increase stress and anxiety in individuals [15].

The findings of this study indicate that the use of 360-degree VR videos during ESWL significantly reduced patients’ levels of pain and anxiety. Similar results are supported by findings in the literature. In a study by Genç et al. (2022), which investigated the effects of VR headsets and stress balls (SB) on pain and vital signs during transrectal prostate biopsy in 96 patients, the use of VR headsets was found to reduce pain levels [29]. Similarly, Candela et al. (2023) reported that the use of VR headsets during ESWL reduced self-reported pain and anxiety levels [30]. Another study also aimed to assess whether a VR device could effectively reduce patient-reported pain during ESWL and reported similar outcomes [28]. Consistent with the findings of the present study, Ketsuwan et al. (2022) investigated the use of immersive virtual reality distraction during flexible cystoscopy and reported a significant reduction in anxiety levels [31]. Similarly, Çelik and Güneş (2025) found that employing a virtual reality headset during ESWL procedures markedly decreased both pain and anxiety [32]. Another study reported that the use of VR headsets during knee arthroscopy decreased patient anxiety and increased satisfaction [33]. However, different results have also been reported in the literature. The study conducted by Quara et al. (2025) demonstrated that use of HypnoVR^®^ during ESWL was clinically safe and feasible; however, it did not result in a significant reduction in pain levels. This finding suggests that the distracting effect of virtual reality may be influenced by factors such as individual perception, stone characteristics, and procedural conditions. Moreover, the retrospective design of the study and the lack of randomization may have limited the ability to fully determine the true effect of the VR intervention [34]. In the present study, all patients received standardized intravenous analgesia in accordance with routine clinical and ethical practice during ESWL. Therefore, the observed effects of VR should be interpreted with this standardized analgesic context. Although both groups benefited from pharmacological pain management, the significantly greater reductions in pain and anxiety observed in the VR group indicate that immersive virtual reality provides an additional therapeutic effect beyond standard analgesia. These findings suggest that VR enhances the overall effectiveness of pain and anxiety management during ESWL.

Upon reviewing the literature, no studies were found that directly investigated the impact of VR use on comfort levels during ESWL. Therefore, comparisons in this discussion were made with studies conducted on similar invasive procedures. In this study, it was observed that patients in the intervention group demonstrated higher levels of comfort. The research findings corroborate the existing literature. Consistent with the findings reported in the literature, Gökçe and Arslan (2023) evaluated the effects of virtual reality and acupressure interventions on pain, anxiety, vital signs, and comfort levels in patients undergoing femoral catheter removal after coronary angiography. Their findings showed that VR significantly reduced pain and anxiety and improved comfort levels during femoral catheter extraction procedures [35]. Similarly, Seçer & Yayla (2025) reported that the use of virtual reality during chest tube removal significantly reduced pain and anxiety levels and improved comfort [36]. It is posited that the virtual headset, owing to the relaxation it induces, mitigates pain and discomfort while enhancing overall comfort. The unique contribution of this study lies in being the first to evaluate the potential effect of VR on comfort during ESWL. Additionally, the findings suggest that VR is a non-pharmacological method that can be integrated into patient care during invasive procedures.

### Study strength and limitations

This study has several strengths. Firstly, the study was not limited to commonly examined variables such as pain and anxiety during ESWL; it also included patient comfort—an outcome that is rarely investigated in the literature—thus offering strong potential for integration into clinical practice. Secondly, a single-blind design was employed. Patients’ levels of pain, anxiety, and comfort were assessed by an independent registered nurse, and data analysis was conducted by an independent biostatistician. This independence minimized potential bias and contributed to the objectivity of the study findings.

However, some limitations must also be acknowledged. The study was carried out at a single center located in the Black Sea Region, which may limit the generalizability of the results to other populations and healthcare settings. Additionally, while subjective scales are valuable for assessing pain, anxiety, and comfort, they may not fully capture the objective physiological dimensions of these experiences. The sample size, although adequate for statistical analysis, was relatively small and may not reflect the broader patient population. Moreover, due to the nature of the intervention, blinding participants was not possible, which may have increased the risk of expectation bias. In addition, because all primary outcomes were self-reported and subjective in nature, the results may have been influenced by participants’ expectations and novelty effects associated with the use of virtual reality, which should be considered when interpreting the findings.

Furthermore, the study evaluated only short-term outcomes immediately after the ESWL procedure and did not include urological and procedural parameters such as stone-free rate, procedural tolerance, need for repeat ESWL sessions, or patient satisfaction. The absence of these clinically relevant outcomes may limit the applicability of the findings in urology- focused clinical settings. Therefore, future studies should incorporate longer follow-up periods and comprehensive urological outcomes. Although a standardized intravenous analgesic protocol was applied to all participants, the study did not evaluate the effect of virtual reality under varying pharmacological regimens. Therefore, the generalizability of the additive clinical benefit of VR across different analgesic protocols may be limited.

## Conclusion

This randomized controlled trial demonstrates that VR is an effective adjunct intervention for reducing pain and anxiety and enhancing comfort during ESWL procedures. The findings highlight the potential of VR as a feasible, safe and non-invasive supportive method that can be integrated into routine nursing care. Despite standardized analgesic administration, VR demonstrated additional benefits in reducing pain and anxiety. However, further multicenter studies with larger samples and longer follow-up periods are required to confirm its long-term clinical benefits and applicability in urological practice.

### Clinical implications

The findings of this study demonstrate that the use of virtual reality headsets during ESWL procedures reduces pain and anxiety levels, thereby enhancing patient comfort. The use of virtual reality headsets appears to be a feasible and well-tolerated approach that may be considered for integration into clinical nursing practices, pending confirmation from larger multicenter studies. This approach is expected to improve patient safety and satisfaction, while also contributing to the advancement of evidence-based practices in surgical nursing.

## Data Availability

The datasets generated and/or analyzed during the current study are available from the corresponding author on reasonable request.

## References

[1] Hill AJ, Basourakos SP, Lewicki P et al (2022) Incidence of Kidney Stones in the United States: The Continuous National Health and Nutrition Examination Survey. J Urol 207:851–856. 10.1097/JU.000000000000233134854755 10.1097/JU.0000000000002331

[2] Eaton SH, Cashy J, Pearl JA et al (2013) Admission rates and costs associated with emergency presentation of urolithiasis: analysis of the Nationwide Emergency Department Sample 2006–2009. J Endourol 27:1535–1538. 10.1089/END.2013.020524251430 10.1089/end.2013.0205PMC3869432

[3] Patti L, Leslie SW (2024) Acute renal colic. In: StatPearls [Internet]. StatPearls Publishing, Treasure Island (FL). Updated 23 Dec2024 URL: https://www.ncbi.nlm.nih.gov/books/NBK431091/28613743

[4] Skolarikos A, Geraghty R, Somani B et al (2025) European Association of Urology Guidelines on the Diagnosis and Treatment of Urolithiasis. Eur Urol 88:64–75. 10.1016/j.eururo.2025.03.01140268592 10.1016/j.eururo.2025.03.011

[5] Wang Z, Feng D, Wei W (2021) Impact of music on anxiety and pain control during extracorporeal shockwave lithotripsy: A protocol for systematic review and meta-analysis. Medicine 100:e23684. 10.1097/MD.000000000002368433530169 10.1097/MD.0000000000023684PMC7850767

[6] Ucer O, Ceylan Y, Ekren F et al (2016) Effect of anxiety and pain on success of shockwave lithotripsy (SWL) for treatment of proximal ureteral and renal pelvic stones. Urolithiasis 44:559–564. 10.1007/S00240-016-0879-427040949 10.1007/s00240-016-0879-4

[7] Gezginci E, Iyigun E, Yalcin S et al (2018) Comparison of Two Different Distraction Methods Affecting the Level of Pain and Anxiety during Extracorporeal Shock Wave Lithotripsy: A Randomized Controlled Trial. Pain Manage Nurs 19:295–302. 10.1016/j.pmn.2017.09.00510.1016/j.pmn.2017.09.00529248604

[8] Hashem A, Ghobrial FK, Elbaset MA et al (2019) Efficacy of pethidine, ketorolac, and lidocaine gel as analgesics for pain control in shockwave lithotripsy: A single-blinded randomized controlled trial. Investig Clin Urol 60:251–257. 10.4111/ICU.2019.60.4.25131294134 10.4111/icu.2019.60.4.251PMC6607066

[9] Takmaz SA, Inan N, Goktug A et al (2008) The Analgesic Effect of 8 and 16 mg Lornoxicam Administered Before Shock Wave Lithotripsy: A Randomized, Double-Blind, Controlled Study. Urology 72:282–285. 10.1016/j.urology.2008.03.03718485457 10.1016/j.urology.2008.03.037

[10] Ahmadnezhad S, Jahromi MSS, Kalani N et al (2022) Pain control in Extracorporeal Shock Wave Lithotripsy (ESWL): A narrative review based on pharmacological and non-pharmacological methods. J Pharm Negat Results 13:89–100. 10.47750/PNR.2022.13.03.014

[11] Özveren H, Faydalı S, Özdemir S (2016) The knowledge and practices of nurses about pain management with non-pharmacological methods. Turkish J Clin Lab 7:99–105. 10.18663/TJCL.286714

[12] Yeniğün SC, Demir Korkmaz F (2025) The Effects of Stress Ball Practice on Patient Anxiety, Pain and Vital Signs During Cataract Surgery: A Randomized Controlled Trial. Pain Manage Nurs 0. 10.1016/j.pmn.2025.04.00910.1016/j.pmn.2025.04.00940399154

[13] Taşçı Ö, Özer N, Çoğaltay N (2024) The Effect of Virtual Reality Application on Pain During Wound Care Dressing Change: A Systematic Review and Meta-analysis of Randomized Controlled Trials. Pain Manage Nurs 25:e99–e107. 10.1016/J.PMN.2023.11.00810.1016/j.pmn.2023.11.00838092603

[14] Hopewell S, Chan AW, Collins GS et al (2025) CONSORT 2025 explanation and elaboration: updated guideline for reporting randomised trials. BMJ 389. 10.1136/BMJ-2024-08112410.1136/bmj-2024-081124PMC1199545240228832

[15] Bozkurt M, Erkoc M, Danis E et al (2022) The Effect of Listening to Music on Reducing Anxiety and Pain During Extracorporeal Shock Wave Lithotripsy; A Randomized Controlled Study. Med Bull Haseki 60:406–410. 10.4274/HASEKI.GALENOS.2022.8728

[16] Gezginci E, Iyigun E, Kibar Y, Bedir S (2018) Three Distraction Methods for Pain Reduction During Cystoscopy: A Randomized Controlled Trial Evaluating the Effects on Pain, Anxiety, and Satisfaction. J Endourol 32:1078–1084. 10.1089/END.2018.049130280915 10.1089/end.2018.0491

[17] Degirmentepe RB, Akca YM, Akman OS et al (2025) The impact of video-animated information on anxiety, satisfaction, and pain perception in patients undergoing ESWL: a randomized controlled study. Urolithiasis 53:1–7. 10.1007/S00240-025-01757-6/TABLES/410.1007/s00240-025-01757-6PMC1203309140285924

[18] Spielberger CD (1970) Manual for the state-trait anxiety inventory. In: Spielberger CD (ed) STAI Manual. Psychologist

[19] Öner N, LeCompte WA (1985) State-Trait Anxiety Inventory Handbook. Boğaziçi University

[20] Price DD, McGrath PA, Rafii A, Buckingham B (1983) The validation of visual analogue scales as ratio scale measures for chronic and experimental pain. Pain 17:45–56. 10.1016/0304-3959(83)90126-46226917 10.1016/0304-3959(83)90126-4

[21] Gift G, Audrey (1989) Visual Analogue Scales: Measurement of Subjective Phenomena. Nursiing Res 38:286–288 2678015

[22] Çelebi D, Yılmaz E, Şahin ST, Baydur H (2020) The effect of music therapy during colonoscopy on pain, anxiety and patient comfort: A randomized controlled trial. Complement Ther Clin Pract 38:101084. 10.1016/J.CTCP.2019.10108432056820 10.1016/j.ctcp.2019.101084

[23] Çavdar AU, Yılmaz E, Baydur H (2020) The Effect of Hand Massage Before Cataract Surgery on Patient Anxiety and Comfort: A Randomized Controlled Study. J PeriAnesthesia Nurs 35:54–59. 10.1016/J.JOPAN.2019.06.01210.1016/j.jopan.2019.06.01231551136

[24] Davey HM, Barratt AL, Butow PN, Deeks JJ (2007) A one-item question with a Likert or Visual Analog Scale adequately measured current anxiety. J Clin Epidemiol 60:356–360. 10.1016/J.JCLINEPI.2006.07.01517346609 10.1016/j.jclinepi.2006.07.015

[25] Youtube (2022) Lauterbrunnen Cennete Yakın /İsviçre 4K /360 / VR VİDEO. https://www.youtube.com/watch?v=MGSALKM2VeI. Accessed 4 Oct 2025

[26] Youtube (2022) Underwater Life, Marsa Alam, Egypt. 360 video in 8K. https://www.youtube.com/watch?v=eKumVFvGHFA. Accessed 4 Oct 2025

[27] Kalaycı Ş (2005) Multivariate Statistical Techniques with SPSS Applied, 6th edn. Asil Publishing Distribution, Ankara

[28] Weynants L, Chys B, D’hulst P et al (2023) Virtual reality for pain control during shock wave lithotripsy: a randomized controlled study. World J Urol 41:589–594. 10.1007/S00345-023-04280-836680576 10.1007/s00345-023-04280-8

[29] Genç H, Korkmaz M, Akkurt A (2022) The Effect of Virtual Reality Glasses and Stress Balls on Pain and Vital Findings During Transrectal Prostate Biopsy: A Randomized Controlled Trial. J PeriAnesthesia Nurs 37:344–350. 10.1016/j.jopan.2021.09.00610.1016/j.jopan.2021.09.00635397973

[30] Candela L, Candela L, Ventimiglia E et al (2023) The Use of a Virtual Reality Device (HypnoVR) During Extracorporeal Shockwave Lithotripsy for Treatment of Urinary Stones: Initial Results of a Clinical Protocol. Urology 175:13–17. 10.1016/j.urology.2023.01.04836796544 10.1016/j.urology.2023.01.048

[31] Ketsuwan C, Matang W, Ratanapornsompong W et al (2022) Prospective randomized controlled trial to evaluate effectiveness of virtual reality to decrease anxiety in office-based flexible cystoscopy patients. World J Urol 40:2575–2581. 10.1007/S00345-022-04142-936048232 10.1007/s00345-022-04142-9

[32] Çelik D, Güneş A (2025) The effect of virtual reality glasses on pain and anxiety during the kidney stone lithotripsy procedure. HEALTH Sci Q 5:233–241. 10.26900/hsq.2712

[33] Sahin G, Basak T (2020) The Effects of Intraoperative Progressive Muscle Relaxation and Virtual Reality Application on Anxiety, Vital Signs, and Satisfaction: A Randomized Controlled Trial. J Perianesthesia Nurs 35:269–276. 10.1016/j.jopan.2019.11.00210.1016/j.jopan.2019.11.00232146074

[34] Quarà A, Candela L, Madden A et al (2025) The use of a virtual reality device (HypnoVR^®^) during extracorporeal shockwave lithotripsy for urinary stones: A case-control study. Fr J Urol 35:102871. 10.1016/J.FJUROL.2025.10287139983906 10.1016/j.fjurol.2025.102871

[35] Gökçe E, Arslan S (2023) Effects of virtual reality and acupressure interventions on pain, anxiety, vital signs and comfort in catheter extraction processes for patients undergoing coronary angiography: A randomized controlled trial. Int J Nurs Pract 29. 10.1111/ijn.1317610.1111/ijn.1317637403339

[36] Seçer H, Yayla A (2025) The effects of virtual reality on pain, anxiety, and comfort during the chest tube removal procedure: A randomized clinical trial. Complement Ther Clin Pract 58:101931. 10.1016/J.CTCP.2024.10193139541838 10.1016/j.ctcp.2024.101931

